# 

**Published:** 1919-01-04

**Authors:** 


### Discharged Officers and Their Outlook.

"Hospital Committee Man" writes to us from Manchester with reference to the article "Discharged Officers and their Outlook" in a recent issue of The Hospital. The letter is too long for our available space. We assure the writer that there is nothing in the nature of drawing comparisons between officers and men in a snobbish spirit. The disabled officer is one of the problems of the war. The disabled man is another. The article only suggests that some of the officers of superior education might be employed with advantage in forms of hospitals and philanthropic work. It is not advocated that disabled officers not fit for secretarial work should be given such work. The writer does not seek to foist useless men on to hospitals, but to bring suitable wounded officers and hospitals together for mutual benefit.—Editor, The Hospital.

### Matrons and Sisters' Appointments.

ROYAL VICTORIA EYE AND EAR HOSPITAL, DUBLIN.—Miss E. M. Power has been appointed matron. She was trained at the Adelaide Hospital, Dublin, and for nine years has held the post of sister at the hospital of which she now becomes matron.

CITY OF WESTMINSTER UNION INFIRMARY, FULHAM ROAD, S.W.—Miss Phœbe Jones has been appointed assistant matron. She was trained at Liverpool Royal Infirmary, later becoming ward and theatre sister there; she has been ward sister under the Metropolitan Asylums' Board, night sister at the British Red Cross Hospital, Netley, and matron of the Newbury District Hospital. She has also been on active service in France. Miss Annie Louisa Wood has been appointed sister. She was trained at the Shirley Warren Infirmary : Southampton, and has been staff nurse at the Norfolk War Hospital.

